# Acute Ascending Thrombosis of Abdominal and Suprarenal Aorta

**DOI:** 10.1155/2014/348064

**Published:** 2014-10-07

**Authors:** Alessandro Robaldo, Stefano Pagliari, Patrizio Colotto

**Affiliations:** Division of Vascular and Endovascular Surgery, Imperia Public Hospital, Via Sant'Agata 57, 18100 Imperia, Italy

## Abstract

We report the diagnostic and successful therapeutic images of an acute occlusion of the abdominal and suprarenal aorta. This lesion is a rare but catastrophic pathology which can cause severe ischemic manifestations, depending on the site of obstruction, with high rate of mortality even after treatment. In the majority of cases it represents a surgical emergency. Although the mechanism of the thrombosis has not been delineated, the proposed etiologies include propagation of thrombus from distal artery occlusion, cardiac thromboembolism, dislodgment of a mural thrombus, or coagulation disorders. Frequent risk factors include advanced atherosclerosis combined with a low flow state because of poor cardiac performance. The management of this condition includes immediate intervention with systemic heparinization, improvement of the cardiac condition, and surgical revascularization based on the clinical and anatomical presentation. In this case the authors highlight the importance of an early detection and early intervention to enhance survival rates and reduce morbidity.

## 1. Case Presentation

**Figure 1:**
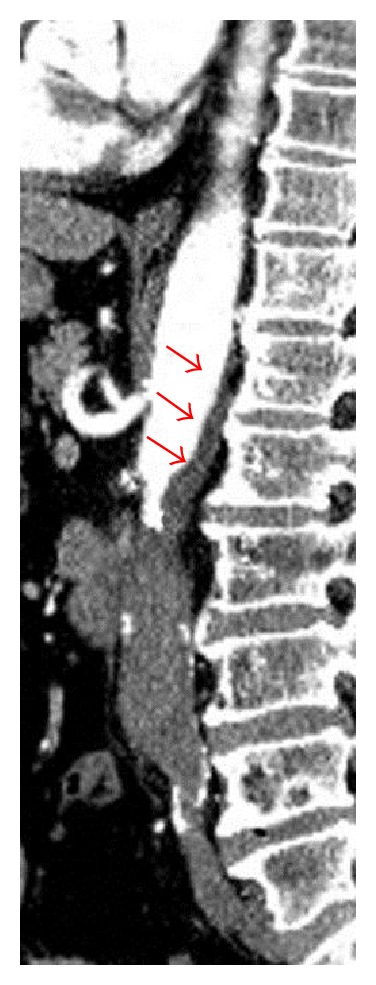
Sagittal view of the acute thrombosis of the abdominal aorta with retrograde partial propagation of the aortic thrombus along the aortic posterior wall up to the level of the patent celiac trunk (red arrows), in addition to severe aortic and iliac calcification.

**Figure 2:**
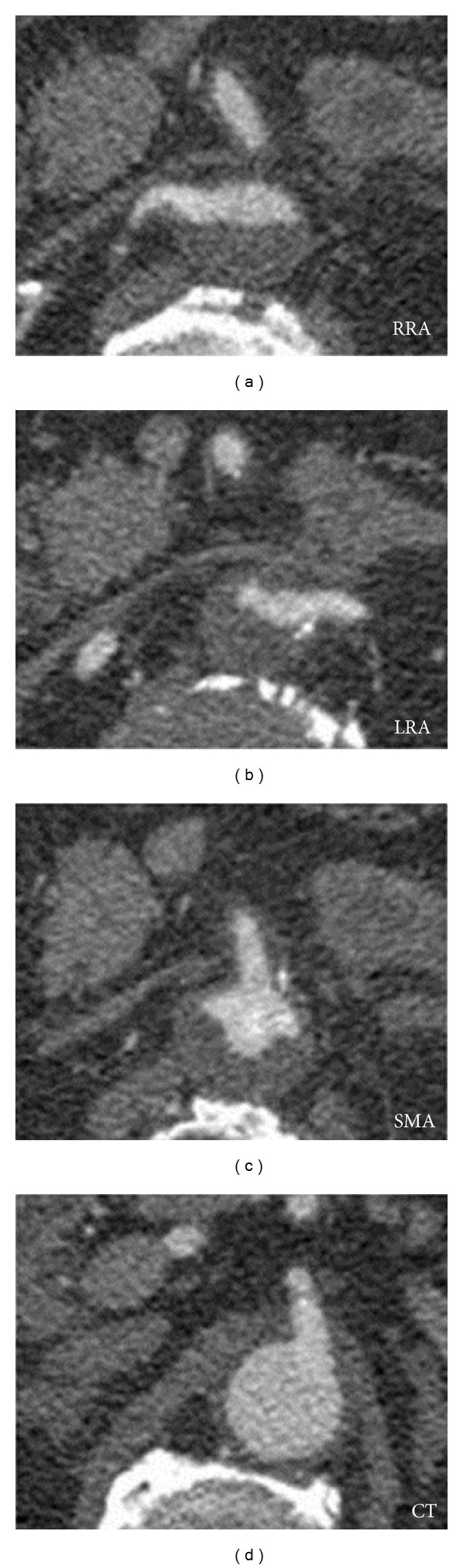
Axial view of the almost complete retrograde thrombosis to the level of the renal arteries and superior mesenteric artery (RRA: right renal artery; LRA: left renal artery; SMA: superior mesenteric artery; CT: celiac trunk).

**Figure 3:**
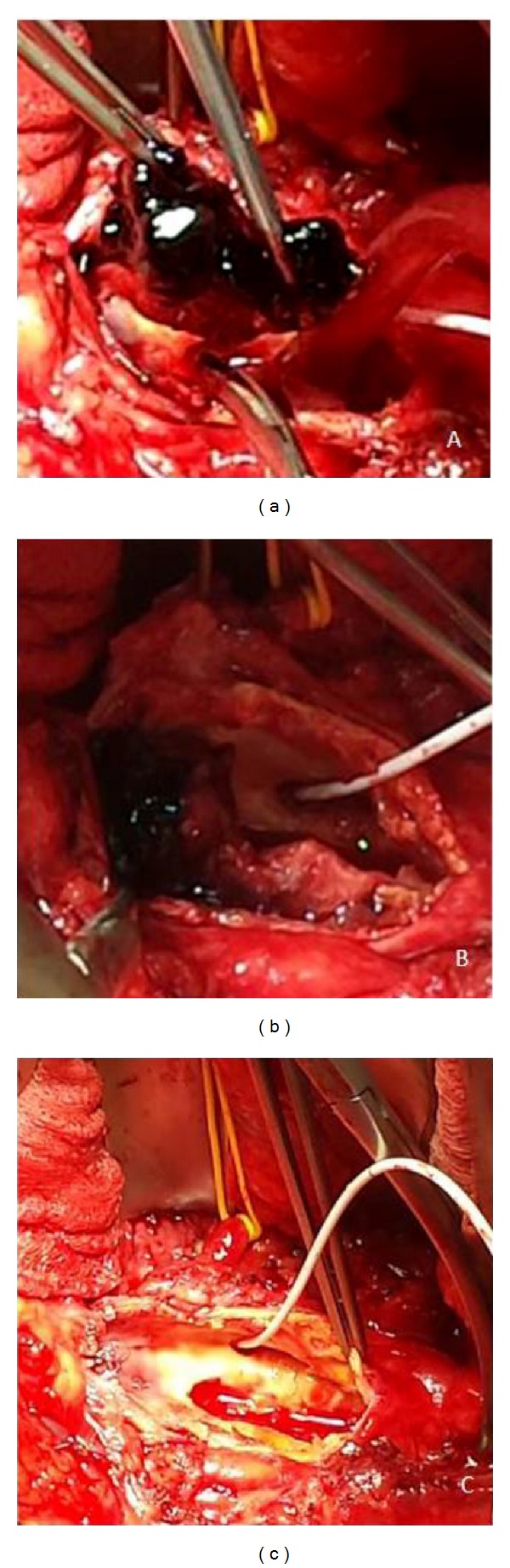
Complete removal of fresh thrombus at the suprarenal aorta level after supraceliac clamping. The yellow vessel loop indicates the left renal artery. The white catheter allowed clamping and perfusing the superior mesenteric artery.

**Figure 4:**
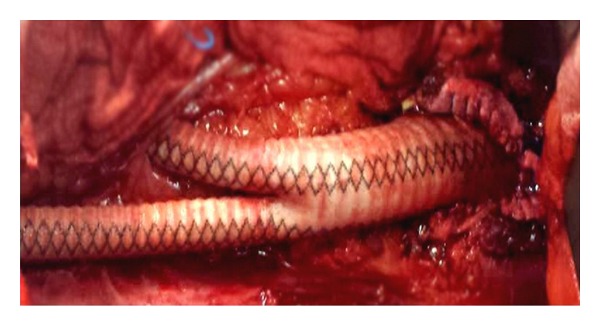
Supraceliac aortofemoral bypass performed through a retroperitoneal approach.

A 71-year-old white male, with a history of heart failure, hypertension, hypercholesterolemia, and major abdominal surgery, was admitted to the emergency room hemodynamically stable with complaint of sudden and severe pain in the lower back and both lower limbs, which was accompanied by lower limb cyanosis, paresthesia, numbness, and progressively paralysis. No recent trauma was reported. Computed tomography (CT) scan revealed occlusion of the infrarenal abdominal aorta and bilateral iliac (including common, external, and internal iliac) arteries with retrograde partial propagation of the aortic thrombus along the aortic posterior wall up to the level of the patent celiac trunk (Figure 1). No aneurysms were detected. Additional findings included severe aortic and iliac calcification with atherosclerotic multistenotic right renal artery and patency of the superior mesenteric artery (Figure 2). Preoperative cardiac testing did not detect atrial fibrillation. Laboratory studies showed elevated ischemia markers with severe metabolic acidosis which progressed even under a continuous intravenous injection of sodium bicarbonate. Due to the proximal extension of the thrombus, retrograde femoral thromboembolectomy was not attempted. Therefore, after systemic heparinization, the patient underwent successful emergency supraceliac aortofemoral bypass performed through a left-flank incision extended into the eleventh intercostal space with retroperitoneal dissection (Figures 3 and 4). After continuous hemodiafiltration in postoperative day one, the patient recovered the reduced urinary output and was discharged 10 days after surgery in good conditions, neurologically intact without renal failure, buttock ischemia, or walking limitation. Postoperative coagulation blood test did not show abnormal values.

## 2. Discussion

Acute aortic occlusion is a rare but catastrophic pathology with high hospital mortality and morbidity even after early recognition and intervention [1]. It may result from thrombus formation, saddle embolism, false-lumen expansion in aortic dissection, aortic trauma, and other etiologies related to arteriosclerosis or hypercoagulability. Specifically, in our case the occlusion was likely caused by an acute thrombosis in a patient with preexisting advanced occlusive disease and a poor cardiac performance due to the known chronic heart failure. The causes of death are associated not only with major organ ischemia but also with severe respiratory failure, fatal arrhythmia, uncontrollable hyperkalemia, or renal failure secondary to myonecrosis. The clinical presentation may vary from acute limb ischemia, neurological symptoms of the lower extremities, abdominal symptoms, and acute hypertension. Clinicians must have a high index of suspicion in patients who present painful paresis or paraplegia. Clinical examination of peripheral pulses in these patients is mandatory [2]. Initial diagnostic test typically includes Doppler Ultrasound and CT-angiography, proved to be important in determining renal and visceral artery involvement [3]. Time is of essence in dealing with those patients and the treatment should move quickly to the operating room. Systemic heparinization, unless contraindicated, immediately after diagnosis and prompt surgical revascularization can reduce the mortality rate. Indeed, this case underlines the importance of an early detection in order to intervene before the ascending thrombosis results in a complete occlusion of all visceral arteries. In case of ascending thrombosis at the level of suprarenal aorta with thrombosis of at least one renal or visceral artery, primary revascularization with either aortofemoral or aortoiliac reconstruction and thrombectomy or bypass of the occluded arteries are the techniques of choice for repair. If no suprarenal aorta involvement is revealed, the initial operation should include an attempt to reestablish inflow by retrograde femoral thromboembolectomy under local anesthesia. If the patient is considered unable to tolerate a major vascular procedure, then axillobifemoral bypass should be performed. Postoperatively, the patient should be aggressively managed to prevent pulmonary and renal complications [4]. Although there has been no consensus in literature, some authors suggest that reperfusion of one or more internal iliac arteries may be a crucial factor in reducing mortality in revascularization treatment in case of concomitant internal iliac artery occlusion [5].
